# Inferred functional potential of microbial communities in traditional African fermented dairy products using 16S rRNA

**DOI:** 10.1099/acmi.0.001182.v4

**Published:** 2026-10-12

**Authors:** David Amedorme, Humphrey Addy, Priscilla Osei-Poku, Alexander Kwarteng

**Affiliations:** 1 Department of Biochemistry and Biotechnology, College of Science, Kwame Nkrumah University of Science and Technology, Kumasi, Ghana; 2 Council for Scientific and Industrial Research, Animal Research Institute, Accra, Ghana; 3 Department of Biomedical Sciences, School of Allied Health Sciences, University of Cape Coast, Cape Coast, Ghana; 4 Kumasi Centre for Collaborative Research in Tropical Medicine, Kwame Nkrumah University of Science and Technology, Kumasi, Ghana

**Keywords:** *Acetobacter*, DNA-helicase, enzymes, *Lactobacillus*, *Streptococcus*

## Abstract

**Background**. Africa is home to several indigenous dairy products that span the continent. These dairy products are widely consumed and cherished for their rich nutritional profile and distinctive taste, shaped by microbial fermentation. However, their microbiome remains largely unexplored, thus limiting knowledge of microbial composition, diversity and functional inference. These limitations have implications for product quality and safety and scientific documentation.

**Methods**. The 16S rRNA dataset on five popularly consumed dairy products, Nono, Wara, Kwerionik, Ghee and Nunu, with the SRA accession number PRJNA532858, was collected for taxonomic classification and diversity analysis using the Quantitative Insights into Microbial Ecology 2 pipeline. Functional inference was conducted using Phylogenetic Investigation of Communities by Reconstruction of Unobserved States 2 version 2.

**Results**. In this dataset, an average of 12 phyla were observed, including *Firmicutes, Proteobacteria, Actinobacteria* and *Bacteroidetes*. At the genus level, lactic acid bacteria, particularly *Lactobacillus* and *Streptococcus*, were predominant in Nono, Wara and Nunu, with relative abundances: 57.7, 33.5 and 39.3% for *Lactobacillus* and 16.6, 36.7 and 39.5% for *Streptococcus*. Functional predictions revealed both shared and product-specific metabolic capabilities, with varying taxonomic contributions to key pathways across samples.

**Conclusion**. Across the analysed samples, the products exhibit both conserved and distinct predicted functional signatures inferred from the 16S rRNA gene data. Particularly, those related to ATP-binding cassette transporters, amino acid metabolism, sugar utilization and biosynthetic pathways. *Streptococcus* dominated in the purine-rich environments of Nono and Nunu; Wara displayed lactic acid bacteria co-dominance, with a focus on peptidoglycan metabolism; Kwerionik exhibited broader microbial diversity, with a three-way taxonomic distribution; and Ghee stood out with *Acetobacter-*driven lipid and oxidative metabolism, showcasing the community’s adaptive plasticity to distinct environmental and processing conditions. These findings suggest that traditional dairy fermentations function as complex microbial ecosystems and highlight their potential importance in food systems and microbial ecology.

## Data summary

The data were collected from the National Center for Biotechnology Information (NCBI), under the accession number PRJNA532858. The samples’ accession numbers are SRX5687335 (Nono), SRX5687308 (Nunu), SRX5687334 (Wara), SRX5687312 (Kwerionik) and SRX5687309 (Ghee). The link to access the data: https://www.ncbi.nlm.nih.gov/bioproject/?term=PRJNA532858.

Original data were published by Diaz *et al*., 2019: Comparison of the microbial composition of African fermented foods using amplicon sequencing. *Scientific reports*, *9* (1), 13863. https://doi.org/10.1038/s41598-019-50190-4.

The individual sequence run accession numbers are: Nono, SRR8901471; Wara, SRR8901472; Kwerionik, SRR8901494; Ghee, SRR8901497 and Nunu, SRR8901498

The combination of keywords for the data search: https://www.ncbi.nlm.nih.gov/bioproject/?term=African+fermented+foods%2C+amplicon+sequencing%2C+and+milk.

All scripts used for the data analysis are available via our GitHub repository: https://github.com/1sed/Fermented-Milk.

## Introduction

Naturally fermented milk products have been a critical component of human nutrition for many years. In Africa, the story is not different. Many African tribes use artisanal fermentation processes to preserve and add value to milk for consumption [1–3]. These tribes hold unique knowledge of these fermented milk products and, as such, are integral to their traditions and cultures. These products are not just a means of enriching the diet but also hold cultural significance for the indigenous tribes who engage in the trade. Although milk is a standard raw material in producing these dairy products, cultural uniqueness and traditional fermentation processes define and confer unique taste, texture and overall product quality on each product [1, 2, 4, 5]. The probiotic organisms found in fermented milk support urogenital health, immune system function, metabolism and gastrointestinal tract balance [6–9]. The leading probiotic carriers in fermented milk are the lactic acid bacteria (LAB). They typically belong to the category of Gram-positive bacteria, including *Lactobacillus, Streptococcus, Pediococcus, Leuconostoc* and *Lactococcus* [3]. The LAB can be inoculated by introducing a starter culture into the raw milk, by natural occurrence under optimal conditions or by pasteurizing the milk [10, 11].

The artisanal process, which relies on the natural introduction of LAB into fermentation, is more common in traditional dairy products produced in Africa. In general, artisanal fermentation is cheap and cost-effective; hence, many of the dairy product producers in developing countries, including countries in Africa, prefer it [12]. No specific standard is applied to fermenting milk when artisanal processes are used. Instead, the producer’s indigenous expertise is employed traditionally [13]. However, because milk has a high nutrient content and an almost neutral pH, improper handling can encourage the uncontrolled proliferation of diverse microbial communities [14]. Invariably, the process of producing dairy products through crude fermentation creates uncertainty about the functioning of the fermentation microbiome and the biological factors that influence it.

In the few studies that have examined microbial content, the methodologies and approaches rely on culture-dependent methods, which are limited in scope. Essentially, the entire microbial community cannot be fully explored using culture-dependent techniques. However, with advances in next-generation sequencing technology and bioinformatics, there have been major strides in understanding the microbiome’s dynamics, structure and function [15, 16]. These tools enable comprehensive studies of the fermentation microbiome in fermented foods. Not just taxonomic classification of culturable and unculturable bacteria, but also functional analysis of metabolic processes [17, 18]. On this basis, this study seeks to explore the inferred functional processes enriched in the fermentation microbiome of selected dairy products. Across the analysed samples, the products exhibit both conserved and distinct predicted functional signatures inferred from the 16S rRNA gene data. Particularly, those related to ABC transporters, amino acid metabolism, sugar utilization and biosynthetic pathways. *Streptococcus* dominated in the purine-rich environments of Nono and Nunu; Wara displayed LAB co-dominance, with a focus on peptidoglycan metabolism; Kwerionik exhibited a broader microbial diversity, characterized by a three-way taxonomic distribution; and Ghee stood out with *Acetobacter-*driven lipid and oxidative metabolism, showcasing the community’s adaptive plasticity to distinct environmental and processing conditions. The findings are descriptive and hypothesis-generating.

## Methods

### Data acquisition

The data were collected from the National Center for Biotechnology Information (NCBI). The search terms include African fermented foods, amplicon sequencing and milk (https://www.ncbi.nlm.nih.gov/bioproject/?term=African+fermented+foods%2C+amplicon+sequencing%2C+and+milk). Per the objectives of this study, the search was limited to these keywords. All data for this study were obtained from the Bioproject PRJNA532858, which was published under Open Access. The article is available under the Creative Commons Attribution 4.0 International License, which permits use, sharing, adaptation, distribution and reproduction in any medium or format, if appropriate credit is given to the original author(s). The project under this Bioproject was funded by the UK Biotechnology and Biological Sciences Research Council (BBSRC) through a Global Challenge Research Fund Data and Resources award, the Institute Strategic Programmes for Food Innovation and Health (BB/R012512/1).

The research under this project (PRJNA532858) used a cross-sectional study design, collecting ∼ 40 fermented food samples produced from raw materials during the workshop, ‘Analysis of the Microbiomes of Naturally Fermented Foods Training Course’. However, this study focuses on fermented dairy products collected from Ghana (Nunu), Nigeria (Nono and Wara) and Uganda (Ghee and Kwerionik). The list of the selected datasets is described in Table 1.

**Table 1.** Selected data description

Accession no.	Run	Country	Library name	Sample name	Condition	DNA concn (ng/μl)
SRX5687335	SRR8901471	Nigeria	Nono	Sample 1	Artisanal	64.6
SRX5687334	SRR8901472	Nigeria	Wara	Sample 2	Artisanal	145.4
SRX5687312	SRR8901494	Uganda	Kwerionik	Sample 3	Artisanal	13.1
SRX5687309	SRR8901497	Uganda	Ghee	Sample 4	Artisanal	44.6
SRX5687308	SRR8901498	Ghana	Nunu	Sample 5	Artisanal	49.4

### DNA extraction and sequencing

According to the published records for accession PRJNA532858, the DNA from the collected samples was obtained after a pre-processing phase. Preprocessing separated the sample’s large, solid particles from the microbial cells. The DNA was extracted using the FastDNA Spin Kit (MP Biomedicals, UK). Using a Qubit 3.0 fluorometer (Invitrogen, Carlsbad, CA), the total amount of DNA extracted from the fermented samples was measured fluorometrically using the Qubit dsDNA BR Assay Kit (Invitrogen). However, when the DNA concentration was below 10 ng/µL, the Qubit dsDNA HS Assay Kit (Invitrogen) was used.

### Bioinformatics analysis

#### Data preprocessing

The FASTQ files used for the quality control had the adapters removed during the preprocessing of the dataset [19]. FastQC was used to assess the quality parameters for downstream analysis. Summary of the FastQC result is presented in Table 2.

**Table 2.** Summary of FastQC results of each product

Filename (paired end)	Total sequence	Total base	Sequences flagged as poor quality	Sequence length
Nono	86,183	17.2 Mbp	0	50–200
Wara	98,058	19.6 Mbp	0	50–200
Kwerionik	81,200	16.2 Mbp	0	32–200
Ghee	80,686	16.1 Mbp	0	39–200
Nunu	89,653	17.9 Mbp	0	33–200

### Quality control and processing

#### Denoising and sequence processing

The paired-end-aligned sequences for both forward and reverse reads were imported into the quantitative insights into microbial ecology 2 (QIIME 2)-amplicon-2024.10 distribution for downstream analysis [20]. The sample datasets were already demultiplexed; therefore, denoising was performed using the Divisive Amplicon Denoising Algorithm 2 (DADA2) plugin in QIIME 2-amplicon to generate representative sequences. The DADA2 plugin generated representative sequences from amplicon sequence variants (ASVs), which were used for taxonomic classification. The choice of the ASV approach, rather than operational taxonomic units, allowed for the inference of exact biological sequences and provided single-nucleotide resolution, enabling the distinction of closely related taxa [21]. Essentially, a more accurate classification was achieved. DADA2 also provided a unique layer of quality assurance by error-correcting, denoising the sequences and outputting amplicon variant sequences [22]. With this, additional filtering was ensured, in addition to the earlier quality control assessment. Details of the DADA2 filtering and quality control metrics are provided in the GitHub repository via: https://github.com/1sed/Fermented-Milk/blob/main/qiime_script.

### Taxonomic classification

To enhance the accuracy of taxonomic classification, the Structured and Improved Large-Scale Alignment database version 138 with clustering level 99% was pre-trained using the QIIME 2 feature-classifier fit-classifier-naive-bayes plugin to generate a customized classification. This approach was adopted based on previous studies that showed that training a Naïve Bayes classifier specifically on the sequenced segment (V4) of target sequences improves the accuracy of 16S rRNA taxonomic classification [23]. Taxonomic assignments were then computed using the QIIME 2 feature-classifier classify-sklearn method. The classifier classify-sklearn method computes taxonomic classification using ASVs. A phyloseq object was created using the classification files (taxonomy.qza, table.qza, rooted tree and metadata file) generated from the QIIME 2 pipeline. The phyloseq object was created in RStudio version 2024.12.0.467 [24]. Raw ASV counts were transformed into relative abundances prior to downstream taxonomic analyses using compositional normalization in R. Specifically, ASV counts for each sample were divided by the total sequencing depth of that sample using the transform_sample_counts() function in the phyloseq package and the transform (‘compositional’) function in the microbiome. The general classification parameters showing the total read counts and the number of ASVs classified under each count are presented in Table 3.

**Table 3.** General statistics on ASV taxa assignment

Taxa	Nono (ASVs)	Wara (ASVs)	Kwerionik (ASVs)	Ghee (ASVs)	Nunu (ASVs)	Uncultured (ASVs)
Phylum	71,381 (9)	79,781 (6)	69,631 (6)	68,972 (5)	76,788 (10)	1
Class	71,381 (13)	79,781 (10)	69,631 (10)	68,972 (9)	76,788 (14)	1
Order	71,381 (31)	79,781 (27)	69,631 (29)	68,972 (26)	76,788 (40)	1
Family	71,381 (43)	79,772 (40)	69,631 (43)	68,972 (37)	76,782 (64)	4
Genus	71,117 (73)	79,463 (69)	64,679 (75)	67,154 (63)	75,774 (119)	20
Species	34,154 (38)	18,109 (45)	19,701 (36)	13,706 (36)	28,912 (59)	80

### Functional inference

Gene content inference was computed using Phylogenetic Investigating of Communities by Reconstructing of Unobserved States 2 (PICRUSt) version 2 [25]. However, because ASVs may not match reference genomes exactly, PICRUSt2 infers functional profiles using hidden-state prediction methods. Evolutionary Placement Algorithm-Next Generation and SATé-enabled Phylogenetic Placement were used first to place ASVs into a reference phylogenetic tree. Then, ASVs were mapped to known genomes to infer functional features. All ASVs generated after denoising (485 ASVs) were successfully retained following phylogenetic placement and Nearest Sequenced Taxon Index (NSTI) prediction and were included in downstream functional analyses. The taxonomy.tsv contained 485 features/ASVs, and the combined-marker-predicted-and-nsti.tsv also contained 485 ASVs successfully retained after phylogenetic placement and NSTI prediction.

Three datasets, namely predicted enzyme (pred-ec-metagenome-unstrat.tsv), predicted Kyoto Encyclopedia of Genes and Genomes (KEGG) genes (pred-metagenome-unstrat.tsv) and predicted enzyme pathways (path-aban-unstrat.tsv) files, were generated and transported for visualization in RStudio version 2024.12.0.467 [24]. Another category of data generated was pred-metagenome-contrib.tsv files from scripts written specifically to stratify functional inference by individual ASVs. These files were generated for pathways, KEGG Orthology (KO) genes and Enzyme Commission. With this, the study traced inferred functions that contribute to ASVs. This approach is known as back-to-feature analysis. Genus-level contributions to each predicted KEGG gene, enzyme function and pathway were calculated as the proportion of the total predicted functional abundance attributed to each taxon within a sample. Outputs are presented as inferred gene-family abundances derived from phylogenetic reconstruction rather than from direct metagenomic sequencing. These values were expressed as percentages to facilitate comparison across samples. The NSTI, which measures the reliability of the PICRUSt2 functional prediction, is presented in Table 4. The analysed data from the five samples fell below 0.06, indicating reliable functional prediction.

**Table 4.** Weighted NSTI scores on the five samples

Sample	Product_name	Weighted_NSTI
Sample 1	Nono	0.025856178
Sample 2	Wara	0.011375733
Sample 3	Kwerionik	0.013936176
Sample 4	Ghee	0.00665794
Sample 5	Nunu	0.023154908

### Phyloseq object creation

To create a unified dataset, a phyloseq object was created using the phyloseq package in R to aggregate the various components of the taxonomic classification datasets. The various datasets, such as the taxonomy.qza, table.qza, the rooted tree and metadata were incorporated into a single dataset. This allows for a unified visualization of the various components of the taxonomic classifications. The phyloseq object also facilitated seamless downstream analysis and taxonomic visualizations, including various forms of bar plots and heatmaps. Taxonomic composition plots were generated in R using ggplot2. Complete scripts, plotting parameters, intermediate processing steps and software dependencies have been provided in the project GitHub repository and README documentation. Other packages used in conjunction with the created phyloseq object include ggplot2, dplyr, vegan, microbiome, pheatmap and stringr. Figures were exported as high-resolution PNG and SVG files (800–1,200 dpi).

## Results

### Core microbial classification

### Dominant genera classification

The genus-level classification based on the top 10 represented genera by relative abundance is presented in Fig. 1. The most represented genera across the five dairy products are *Lactobacillus* at (32.1%, *n*=22,117), (35.1%, *n*=24,458), (57.7%, *n*=41,176), (39.3%, *n*=30,203) and (33.5%, *n*=26,689) for Ghee, Kwerionik, Nono, Nunu and Wara, respectively. In Ghee and Kwerionik, *Acetobacter* ranks second at 30.2% (*n*=20,840) and 15.5% (*n*=10,802), respectively. However, *Streptococcus* is the second most dominant genus at (16.6%, *n*=11,837), (39.5%, *n*=30,324) and (36.7%, *n*=29,245) for Nono, Nunu and Wara, respectively. *Lueconostoc* is one of the dominant genus categories in Kwerionik (9.8%, *n*=6,843) and Nunu (2.7%, *n*=4,041). *Weissella* is present in all five dairy products at (4.3%, *n*=2,970), (3.3%, *n*=2,332), (5.8%, *n*=4,155), (0.9%, *n*=703) and (8.8%, *n*=7,032), respectively.

**Fig. 1.**
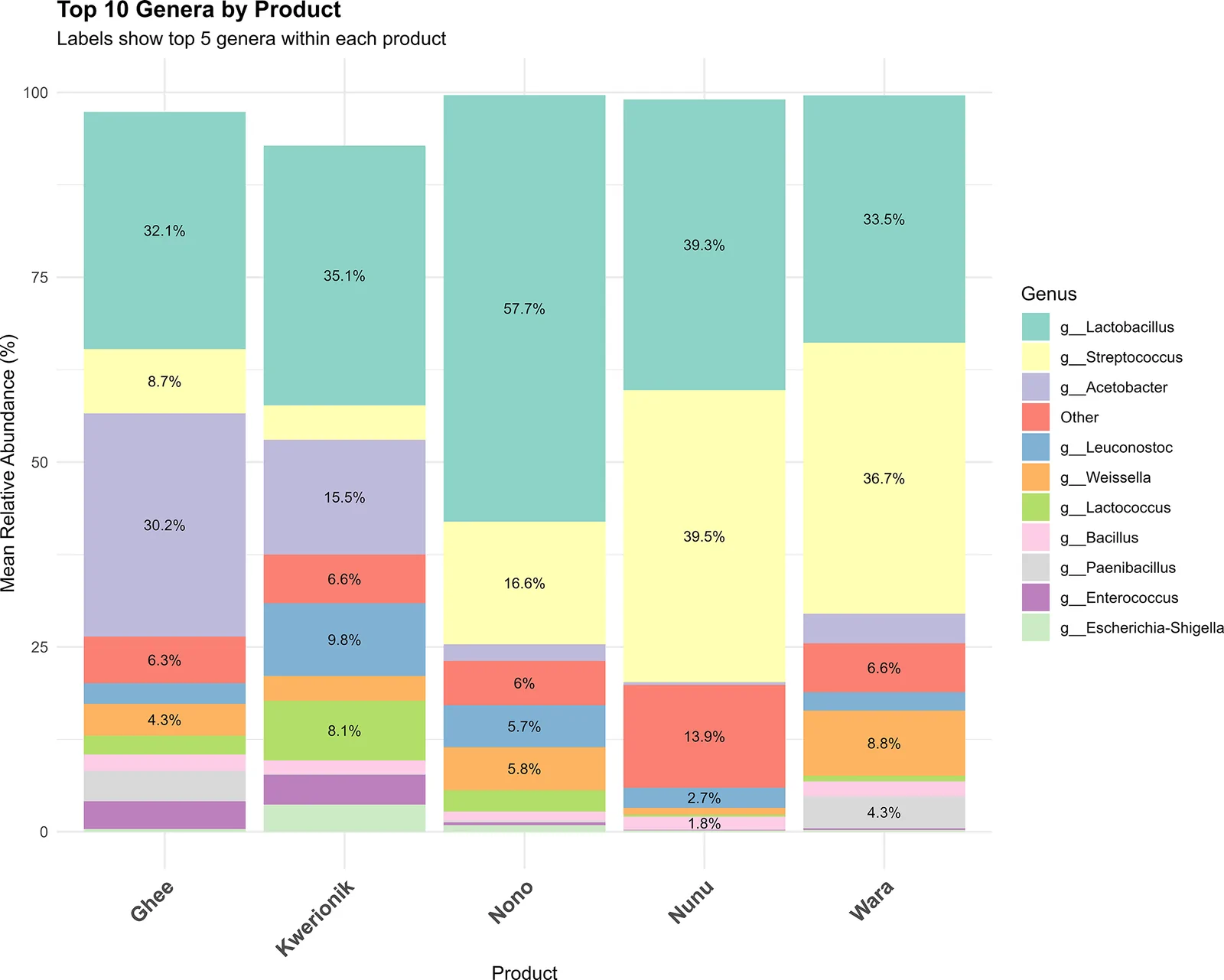
Dominant genera identified in five dairy products. The stacked bar chart represents the proportion of quality-controlled sequencing reads assigned to each bacterial genus relative to the total number of quality-controlled reads in each sample.

### Alpha diversity analysis at genus level

Fig. 2 presents genus-level alpha diversity varying across the five fermented dairy products, as shown by the Shannon and Simpson indices. Kwerionik recorded the highest Shannon diversity (2.13), followed by Ghee (2.01), Wara (1.80), Nunu (1.79) and Nono (1.61). A similar pattern was observed for the Simpson index, with Kwerionik showing the highest value (0.80), followed by Ghee (0.78), Wara (0.74), Nunu (0.68) and Nono (0.63). These results suggest that Kwerionik had the most even and diverse genus composition, whereas Nono showed lower diversity and stronger dominance by fewer taxa. Because each product was represented by a single sample, these indices are interpreted descriptively.

**Fig. 2.**
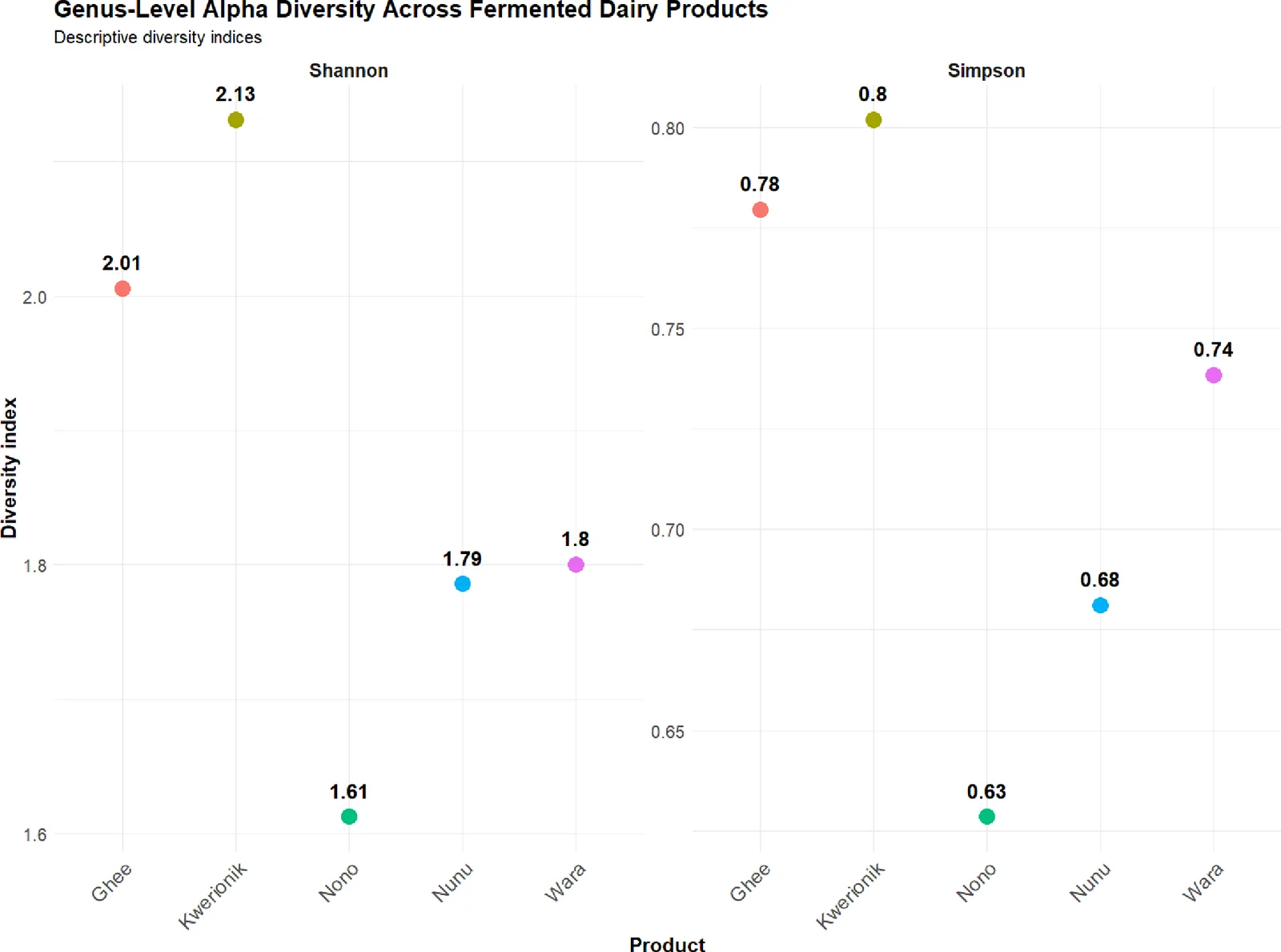
Genus-level alpha diversity of the fermented dairy products, as shown by the Shannon and Simpson indices.

#### Functionally abundant enzymes at the genus level

The clustered heatmap in Fig. 3 displays the top 10 comparatively abundant functional predictions by enzyme activity of the dairy products’ fermentation microbiota. Nunu had the highest functional capacities for enzyme-catalysed enzymatic reactions, site-specific DNA methyltransferase (adenine-specific) (EC:2.1.1.72), DNA 5′−3′ helicase-DNA 3′−5′ helicase (EC:3.6.4.12), DNA-directed RNA polymerase (EC:2.7.7.6), DNA-directed DNA polymerase (EC:2.7.7.7), histidine kinase (EC:2.7.13.3) and serine-type d-Ala-d-Ala carboxypeptidase (EC:3.4.16.4) at relative contributions of 22.4, 21.9, 22.3, 22.4 and 23.8%, respectively. Wara also recorded the highest relative contribution for 6-phospho-β-glucosidase (EC:3.2.1.86), ABC-type polar-amino-acid transporter (EC:3.6.3.21), asparaginyl-tRNA synthase (glutamine-hydrolysing) (EC:6.3.5.6), and glutaminyl-tRNA synthase (glutamine-hydrolysing) (EC:6.3.5.7) at relative contributions of 28.2, 26.6, 21.6 and 21.6%, respectively.

**Fig. 3.**
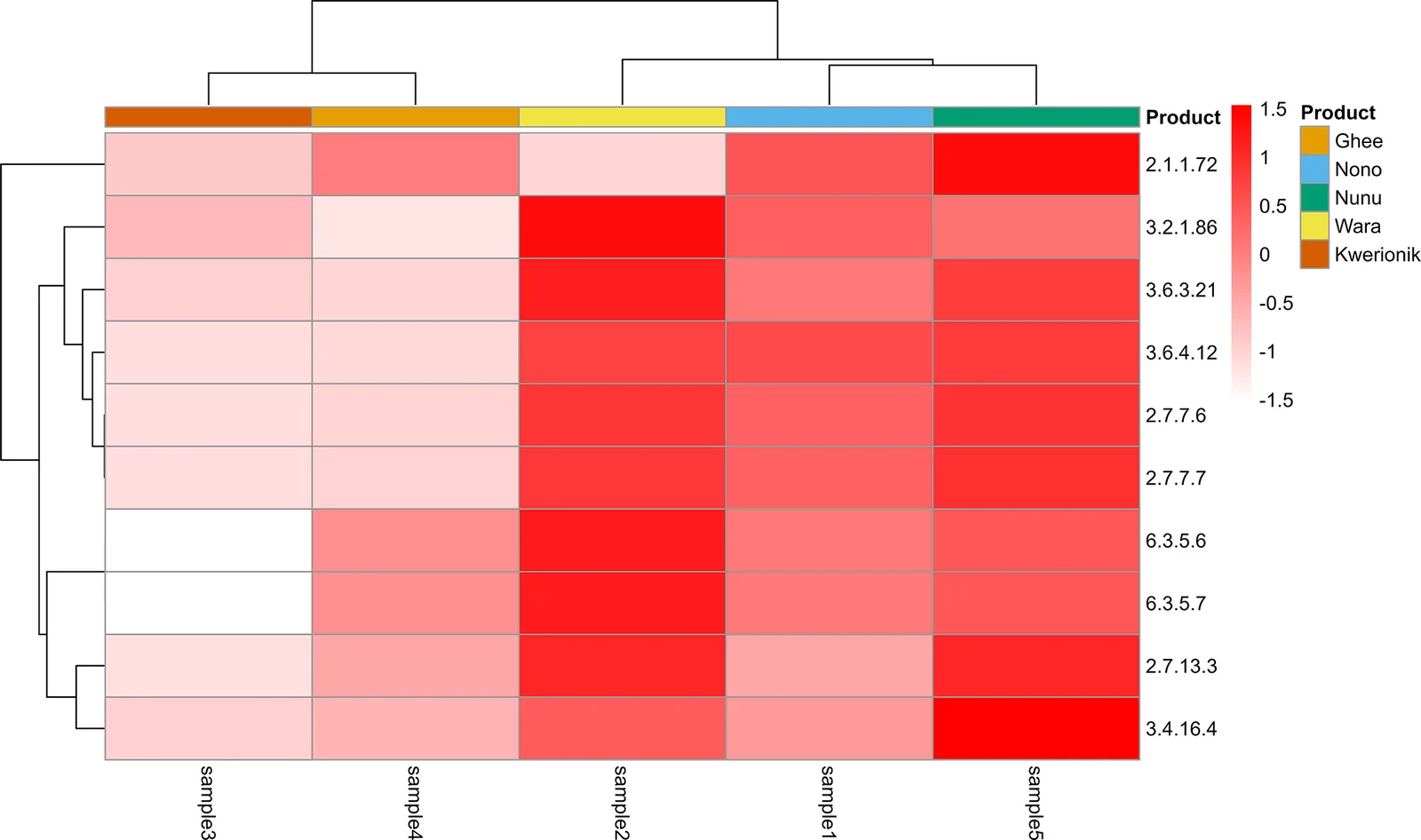
Predicted functional activity for enzymatic reactions for the five dairy products. EC:2.1.1.72=site-specific DNA-methyltransferase (adenine-specific), EC:3.6.4.12=DNA-helicase, EC:2.7.7.6 = DNA-directed RNA polymerase, EC:2.7.7.7 = DNA-directed DNA polymerase, EC:2.7.13.3=histidine kinase, EC:3.4.16.4=serine type d-Ala-d-Ala carboxypeptidase, EC:3.2.1.86=6-phospho-β-glucosidase, EC:3.6.3.21=ABC type polar-amino-acid transporter, EC:6.3.5.6 = asparaginyl tRNA synthase (glutamine-hydrolysing) and EC:6.3.5.7 = glutaminyl tRNA synthase (glutamine-hydrolysing).

### Functionally abundant KO genes at the genus level

The top functionally abundant KO genes in the fermentation microbiome of the dairy products are presented in Fig. 4. Nunu and Wara appear to be more functionally enriched by the abundance of KEGG genes comparatively. Specifically, Nunu has more abundant ABC-2 type transport system permease protein (K01992), ABC-2 type transport system ATP-binding protein (K01990) and ATP-binding cassette (ABC), subfamily B, bacterial (K06147) knockout genes at relative contributions of 29.3, 26.3 and 26.9%, respectively. Wara is also more enriched with ABC, subfamily C, bacterial (K06148), polar amino acid transport system ATP-binding protein (K02028), putative ABC transport system permease protein (K02004), polar amino acid transport system permease protein (K02029), putative ABC transport system ATP-binding protein (K02003) and polar amino acid transport system substrate-binding protein (K02030), at relative contributions of 26.3, 26.6, 26.2, 25.8, 27.3 and 26.1%, respectively.

**Fig. 4.**
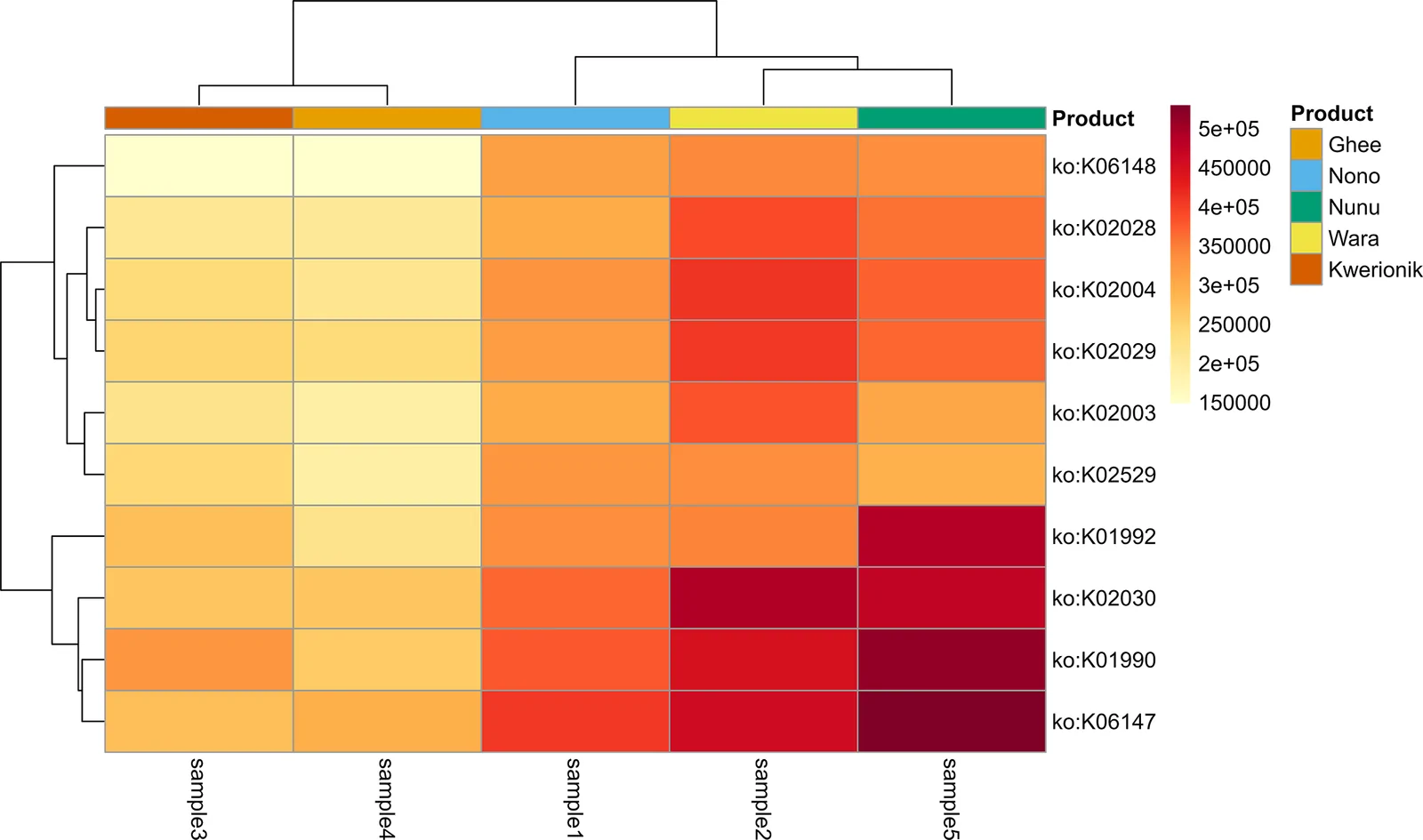
Predicted functional activity for KO genes for the five dairy products. K01992=ABC-2 type transport system permease protein (K01992), ABC-2 type transport system ATP-binding protein, K01990=ABC-2 type transport system ATP-binding protein, K06147=ABC, subfamily B, bacterial, K06148=ABC subfamily C, bacterial, K02028=polar amino acid transport system ATP-binding protein, K02004=putative ABC transport system permease protein, K02029=polar amino acid transport system permease protein, K02003=putative ABC transport system ATP-binding protein and K02030=polar amino acid transport system substrate-binding protein.

### Functionally abundant pathways at the genus level

Fig. 5 presents the top 10 abundant pathways for the five dairy products. In all five dairy products, the most abundant pathway is PWY0-1586 (UMP biosynthesis I). However, Nunu observed the highest relative contribution, PWY0-1586 (UMP biosynthesis I) at 24.6%, followed by Wara dairy product at 23.1%, and the lowest relative contribution was recorded for Kwerionik at 16.1%. Pathways, guanosine deoxyribonucleotides *de novo* biosynthesis II (PWY-7222) and adenosine deoxyribonucleotides *de novo* biosynthesis II (PWY-7220) appear to have similar abundances.

**Fig. 5.**
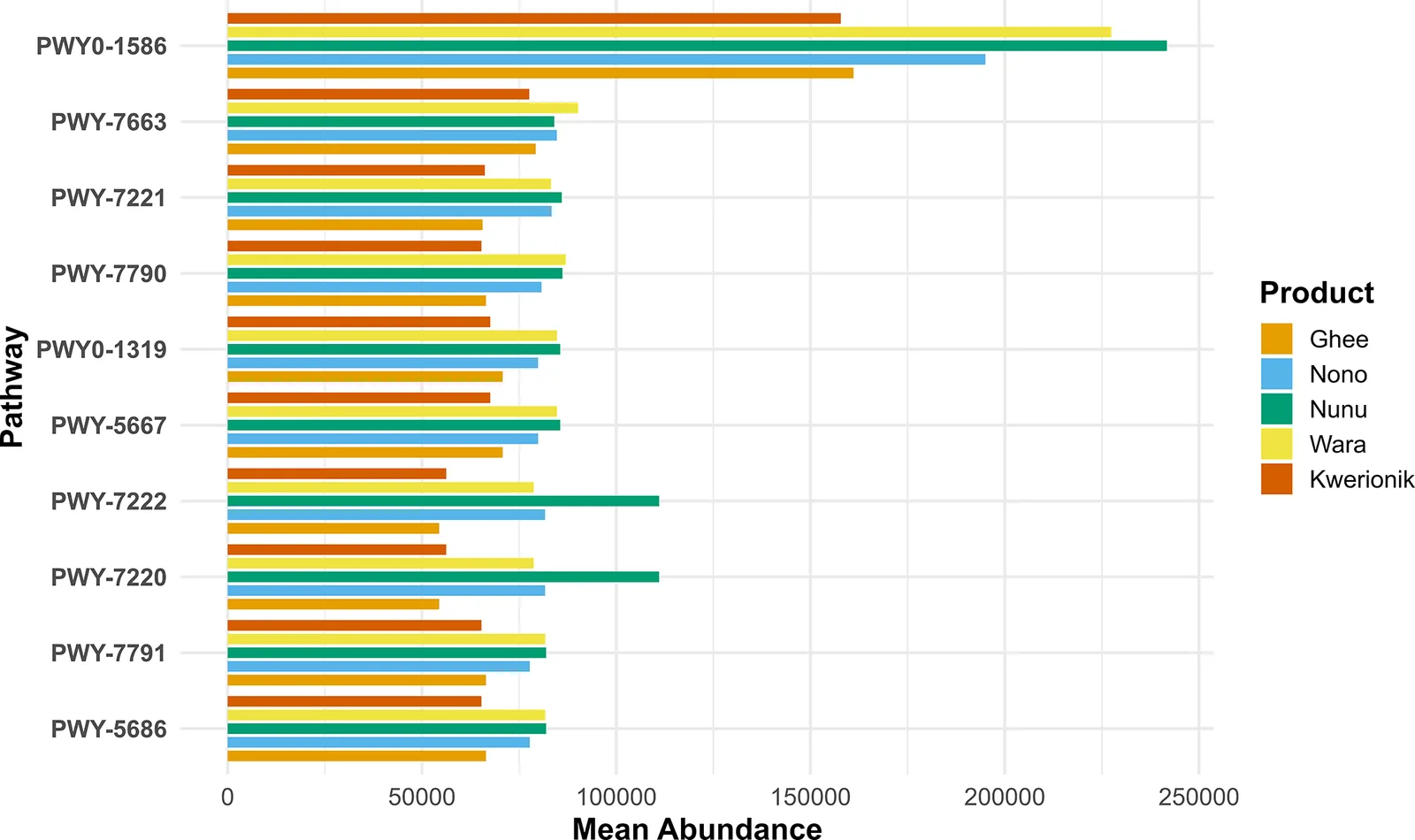
Predicted abundant pathways for the five dairy products. PWY-7220=adenosine deoxyribonucleotides *de novo* biosynthesis II, PWY-7222=guanosine deoxyribonucleotides *de novo* biosynthesis II, PWY0−1319=CDP-diacylglycerol biosynthesis II, PWY-5667=CDP-diacylglycerol biosynthesis I, PWY-1586=peptidoglycan maturation (meso-diaminopimelate containing), PWY-7221=guanosine ribonucleotides *de novo* biosynthesis, PWY-7790=UMP biosynthesis II, PWY-5686=UMP biosynthesis I, PWY-7791=UMP biosynthesis III and PWY-7663=gondoate biosynthesis (anaerobic).

### Individual product level functional analysis by genus

#### Genus contribution to the enriched enzymes in Nono, Wara and Nunu

The genus-level contributions to the top-predicted enzyme-associated functional features in Nono, Wara and Nunu are shown in Fig. 6. The genus *Lactobacillus* accounts for the top predicted enzymatic reactions enriched in the fermentation of Nono through the enzymes, site-specific DNA-methyltransferase (adenine-specific) (EC 2.1.1.72), histidine kinase (2.7.13.3), DNA-directed RNA polymerase (EC 2.7.7.6) and DNA-directed DNA polymerase (2.7.7.7). *Lactobacillus* predicted functional abundances are 57.9, 49.1, 61.3 and 61.2%, respectively.

**Fig. 6.**
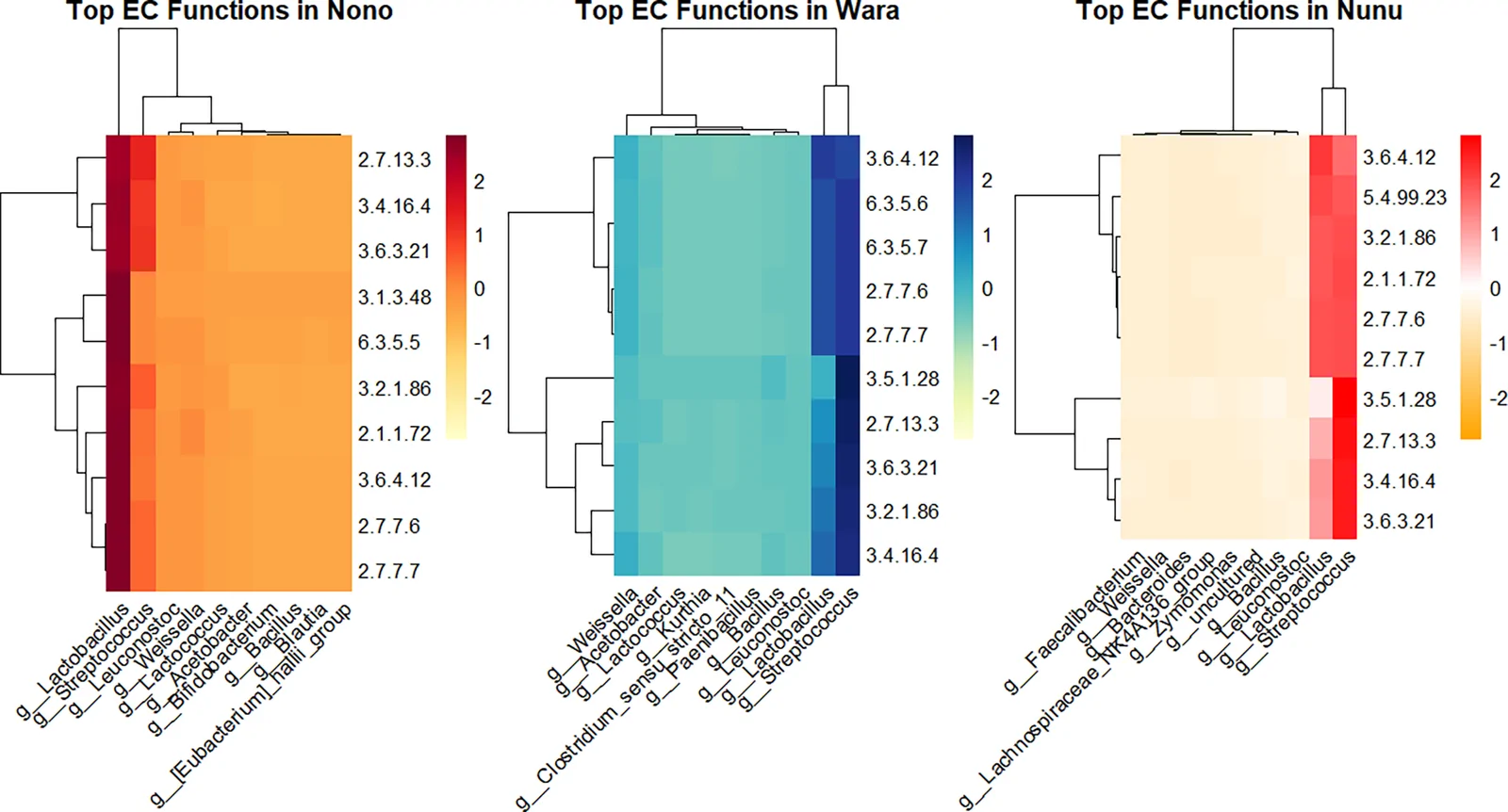
Predicted functional activity for enzymatic reactions for Nono, Wara and Nunu dairy products. EC:2.1.1.72=site-specific DNA-methyltransferase (adenine-specific), EC:3.6.4.12=DNA-helicase, EC:2.7.7.6 = DNA-directed RNA polymerase, EC:2.7.7.7 = DNA-directed DNA polymerase, EC:2.7.13.3=histidine kinase, EC:3.4.16.4=serine type d-Ala-d-Ala carboxypeptidase, EC:3.2.1.86=6-phospho-β-glucosidase, EC:3.6.3.21=ABC type polar-amino-acid transporter, EC:3.1.3.48=protein-tyrosine-phosphatase, EC:6.3.5.5=carbamoyl-phosphate synthase (glutamine-hydrolysing), EC:6.3.5.6=asparaginyl tRNA synthase (glutamine-hydrolysing), EC:6.3.5.7=glutaminyl tRNA synthase (glutamine-hydrolysing), EC:3.5.1.28=*N*-acetylmuramoyl-l-alanine amidase and EC:5.4.99.23=23S rRNA pseudouridine1911/1915/1917 synthase.

In Wara, *Streptococcus* was the dominant contributor to several enriched predicted enzyme-associated functions, including *N*-acetylmuramoyl-l-alanine amidase (EC 3.5.1.28), ABC-type polar amino acid transporter (EC 3.6.3.21) and histidine kinase (EC 2.7.13.3), accounting for 70.7, 59.6 and 58.2% of the total predicted functional abundances for these enzymes, respectively. *Lactobacillus* also contributed 39.4% to the enzyme DNA helicase (EC 3.6.4.12) in Wara. The most abundant enzyme activities in Nunu are catalysed by the enzymes *N*-acetylmuramoyl-l-alanine amidase (EC 3.5.1.28), histidine kinase (EC 2.7.13.3), serine-type d-Ala-d-Ala carboxypeptidase (EC 3.4.16.4) and ABC-type polar-amino-acid transporter (EC 3.6.3.21), which are accounted for by *Streptococcus* at 74.8, 62.8, 55.9 and 61.0%. *Lactobacillus* was the dominant contributor to the inferred functional potential for DNA helicase (EC 3.6.4.12) and pseudouridine synthase (EC 5.4.99.23), contributing 49.6 and 47.1% of the total predicted abundances for these enzyme functions, respectively. These values reflect the relative contribution of taxa to predicted enzyme-associated functions rather than direct measurements of enzyme activity.

### Genus contribution to the enriched enzymes in Kwerionik and Ghee

The genus composition regulating the top functional predictions by enzyme activity in Kwerionik and Ghee dairy products is presented in Fig. 7. *Lactobacillus* was the dominant contributor to several enriched predicted enzyme-associated functions in Kwerionik. The most represented enzymes included serine-type d-Ala-d-Ala carboxypeptidase (EC 3.4.16.4), DNA helicase (EC 3.6.4.12), asparaginyl-tRNA synthase (glutamine-hydrolysing) (EC 6.3.5.6), DNA-directed DNA polymerase (EC 2.7.7.7) and 6-phospho-β-glucosidase (EC 3.2.1.86), with *Lactobacillus* contributing 45.9, 42.3%, 41.9, 40.4 and 39.8% of the total predicted functional abundances for these enzymes, respectively. Other top-represented predicted enzyme-associated functions included DNA-directed RNA polymerase (EC 2.7.7.6), ABC-type polar amino acid transporter (EC 3.6.3.21) and alcohol dehydrogenase (EC 1.1.1.1).

**Fig. 7.**
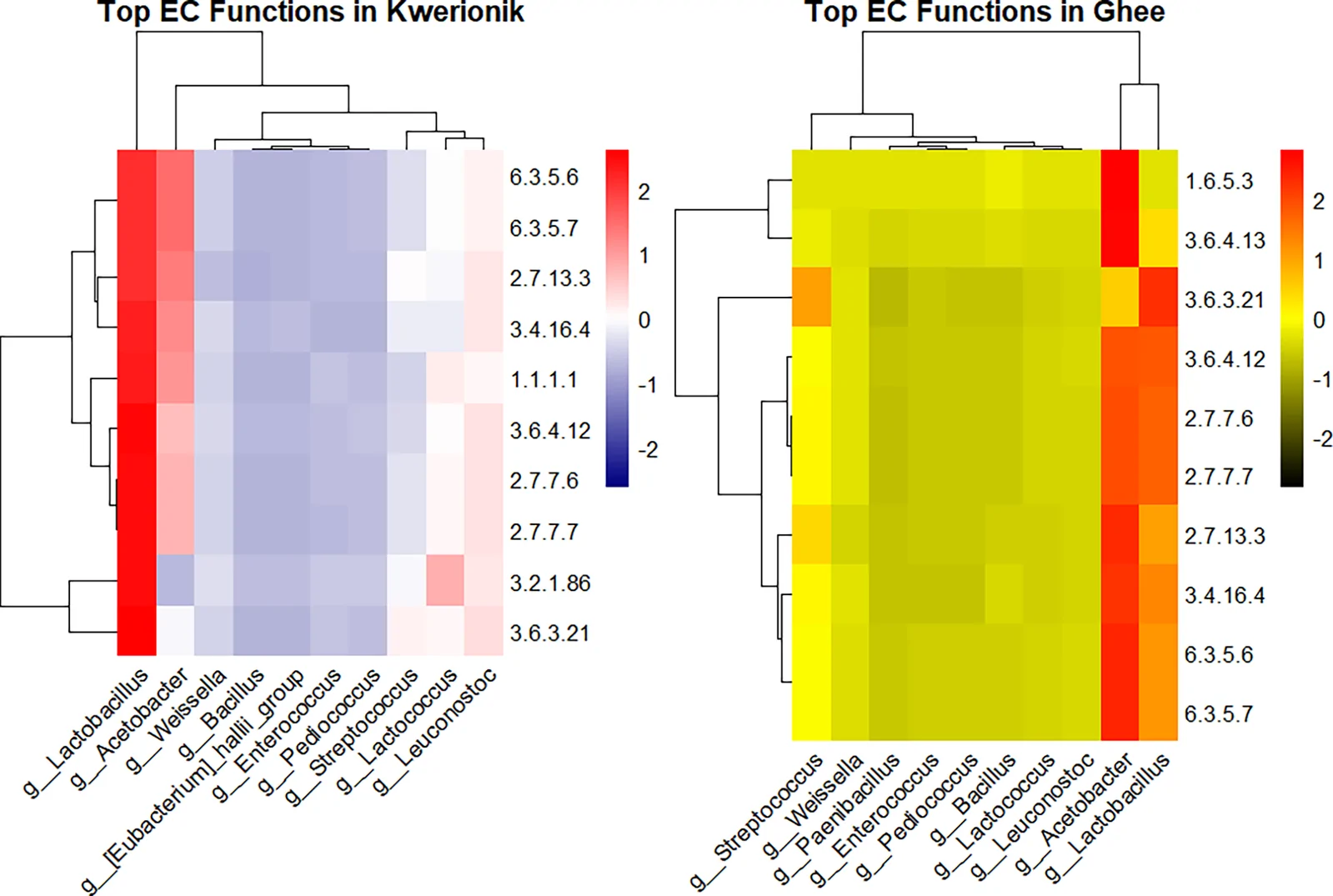
Predicted functional activity for enzymatic reactions for the Ghee dairy product. EC:3.6.4.12=DNA-helicase, EC:2.7.7.6=DNA-directed RNA polymerase, EC:2.7.7.7=DNA-directed DNA polymerase EC:2.7.13.3=histidine kinase, EC:3.4.16.4=serine type d-Ala-d-Ala carboxypeptidase, EC:3.6.3.21=ABC type polar-amino-acid transporter, EC:6.3.5.6=asparaginyl tRNA synthase (glutamine-hydrolysing), EC:6.3.5.7=glutaminyl tRNA synthase (glutamine-hydrolysing), EC:1.6.5.3=NADH:ubiquinone reductase, EC:3.6.4.13=RNA helicase. EC:3.2.1.86=6-phospho-β-glucosidase, EC:1.1.1.1=alcohol dehydrogenase.

In Ghee, *Acetobacter* was the dominant contributor to several enriched predicted enzyme functions, including NADH: ubiquinone reductase (EC 1.6.5.3), RNA helicase (EC 3.6.4.13), asparaginyl-tRNA synthase (glutamine-hydrolysing) (EC 6.3.5.6), glutaminyl-tRNA synthase (glutamine-hydrolysing) (EC 6.3.5.7), DNA helicase (EC 3.6.4.12), DNA-directed RNA polymerase (EC 2.7.7.6), DNA-directed DNA polymerase (EC 2.7.7.7) and histidine kinase (EC 2.7.13.3), contributing 96.2, 65.7, 47.2, 47.2, 36.3, 36.3, 36.7 and 43.7% of the total predicted functional abundances for these enzymes, respectively.

### Genus contribution to the enriched KEGG genes in Nono, Wara and Nunu

Fig. 8 presents the top predicted functionally abundant KO genes in the fermentation microbiome of the Nono, Wara and Nunu dairy products. *Lactobacillus* contributed the most to all the top-represented KEGG genes, with top contributions being putative ABC transport system ATP-binding protein (K02003), LacI family transcriptional regulator, galactose operon repressor (K02529), ABC, subfamily B, bacterial (K06147) and ABC, subfamily C, bacterial (K06148) at 57.6, 67.2, 56.8 and 73.4%, respectively. *Streptococcus* also contributed substantially, ranging between 13.0 and 33.02%, to all the top-represented KEGG genes.

**Fig. 8.**
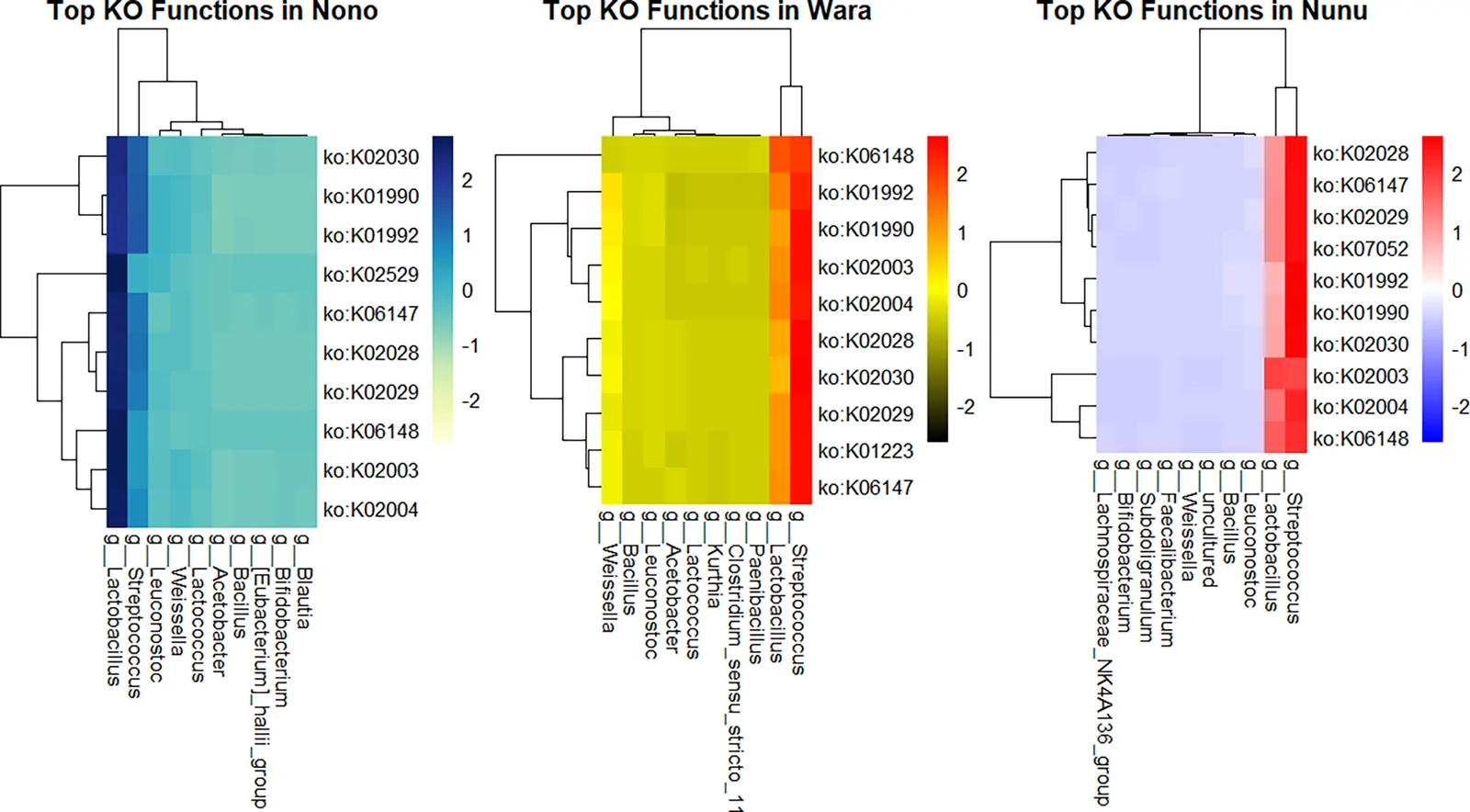
Predicted functional activity for KO genes for the dairy products. Rel_abundance=(abundance of a specific KO contributed by a genus)/(sum of all KO abundances across all genera and KOs)×100. K01992=ABC-2 type transport system permease protein, K01990=ABC-2 type transport system ATP-binding protein, K06147=ABC, subfamily B, bacterial, K06148=ABC, subfamily C, bacterial, K02028=polar amino acid transport system ATP-binding protein, K02004=putative ABC transport system permease protein, K02029=polar amino acid transport system permease protein, K02003=putative ABC transport system ATP-binding protein, K02030=polar amino acid transport system substrate-binding protein, K02529=LacI family transcriptional regulator, galactose operon repressor. K01223=6-phospho-beta-glucosidase. K07052=CAAX protease family protein.

In Wara, *Streptococcus* contributed the most to the top-represented KEGG genes, including putative ABC transport system ATP-binding protein (K02003), polar amino acid transport system ATP-binding protein (K02028), polar amino acid transport system permease protein (K02029), polar amino acid transport system substrate-binding protein (K02030) and ABC, subfamily B, bacterial (K06147) at 51.8, 59.6, 57.5, 70.0 and 54.3% respectively. *Lactobacillus* also contributed substantially, ranging from 23.8 to 45.4%, to the top-represented KEGG genes.

In Nunu, *Streptococcus* contributed the most to all the top-represented KEGG genes, ATP-binding protein (K01990), ABC-2 type transport system permease protein (K01992), putative ABC transport system permease protein (K02004), polar amino acid transport system ATP-binding protein (K02028), polar amino acid transport system permease protein (K02029), polar amino acid transport system substrate-binding protein (K02030), and ABC, subfamily B, bacterial (K06147) at 62.6, 65.7, 52.6, 60.8, 59.7, 65.2 and 60.0%, respectively. *Lactobacillus* also contributed the most to the KEGG gene, putative ABC transport system ATP-binding protein (K02003) at 43.6%.

### Genus contribution to the enriched KEGG genes in Kwerionik and Ghee

Fig. 9 presents the top predicted functionally abundant KO genes in the fermentation microbiome of the Kwerionik and Ghee dairy products. *Lactobacillus* contributed the most to all the top-represented KEGG genes with top contributions being putative ABC transport system ATP-binding protein (K02003), putative ABC transport system permease protein (K02004), LacI family transcriptional regulator, galactose operon repressor (K02529), ABC, subfamily B, bacterial (K06147) and amino acid transporter, AAT family (K03293) at 45.6, 46.5, 52.2, 56.8 and 42.3%, respectively. *Lactococcus* and *Leuconostoc* also contributed substantially, ranging from 7.4 to 18.3% and from 5.2 to 22.7%, respectively, to the top-represented KEGG genes.

**Fig. 9.**
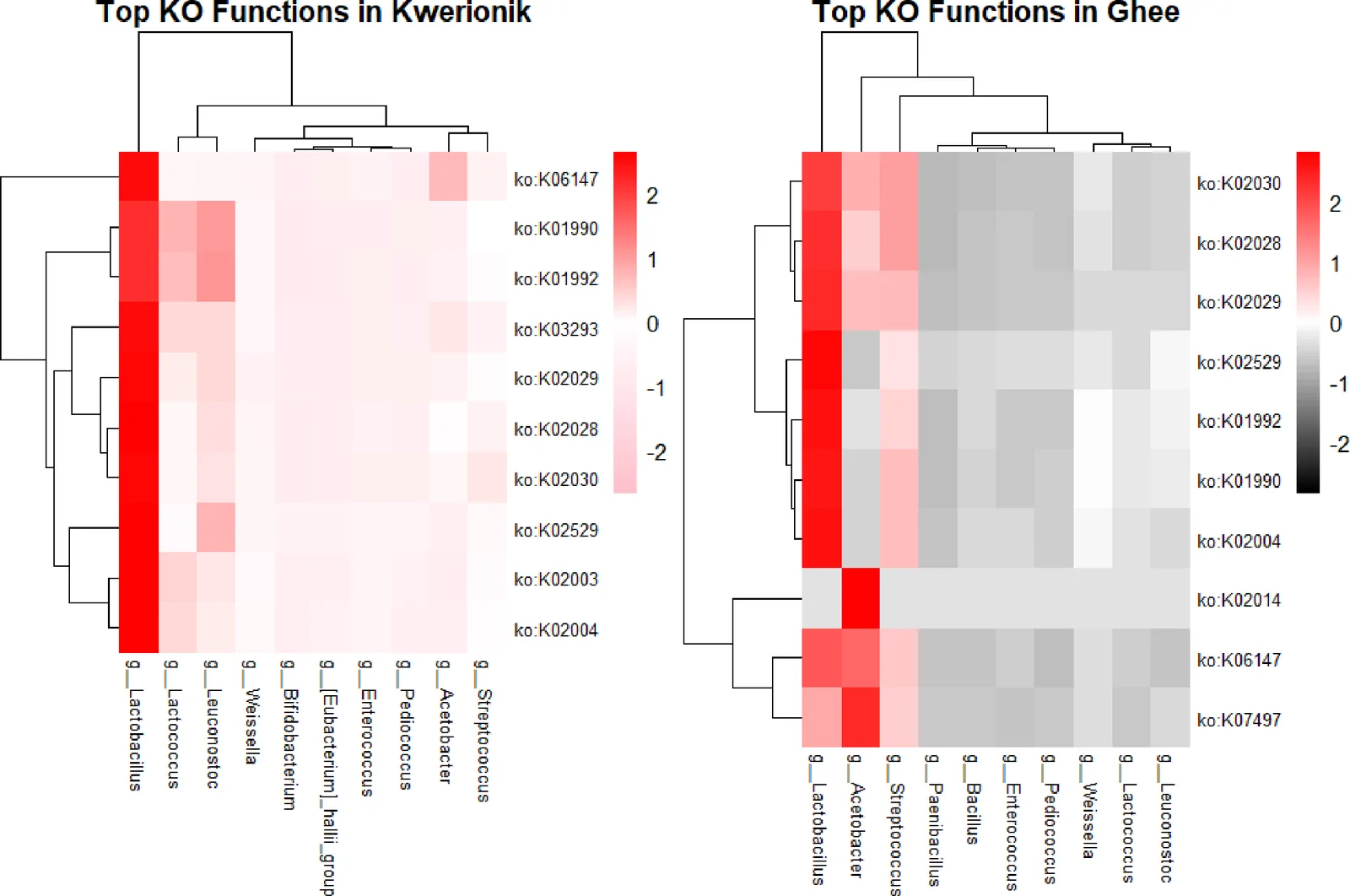
Predicted functional activity for KO genes for the Kwerionik dairy product. Rel_abundance=(abundance of a specific KO contributed by a genus)/(sum of all KO abundances across all genera and KOs)×100. K01992=ABC-2 type transport system permease protein, K01990=ABC-2 type transport system ATP-binding protein, K06147=ABC, subfamily B, bacterial, K06148=ABC, subfamily C, bacterial, K02028=polar amino acid transport system ATP-binding protein, K02004=putative ABC transport system permease protein, K02029=polar amino acid transport system permease protein, K02003=putative ABC transport system ATP-binding protein, K02030=polar amino acid transport system substrate-binding protein, K03293=amino acid transporter, AAT family, K07497=putative transposase.

In Ghee, *Lactobacillus* contributed the most to all the top-represented KEGG genes with top contributions being ATP-binding protein (K01990), ABC-2 type transport system permease protein (K01992), putative ABC transport system permease protein (K02004), polar amino acid transport system permease protein (K02029) and LacI family transcriptional regulator, galactose operon repressor (K02529) at 41.9, 41.2, 48.8, 42.2 and 57.7%, respectively. *Acetobacter* also contributed the most to the KEGG genes, putative ABC transport system permease protein (K02014) and putative transposase (K07497) at 99.2 and 45.4%, respectively.

### Genus contribution to the enriched pathways in Nono

Fig. 10 presents the top predicted functionally abundant metabolic pathways in the fermentation microbiome of the Nono dairy product. *Lactobacillus* contributed the most to all the top-represented pathways, UMP biosynthesis I (PWY-5686), UMP biosynthesis III (PWY-7791), CDP-diacylglycerol biosynthesis II (PWY0-1319), CDP-diacylglycerol biosynthesis I (PWY-5667) and peptidoglycan maturation (meso-diaminopimelate containing) (PWY0-1586) at 61.9, 64.3, 64.6, 64.6 and 50.4%, respectively. *Streptococcus* also contributed substantially, ranging from 13.0 to30.0% across the top-represented enzyme pathways.

**Fig. 10.**
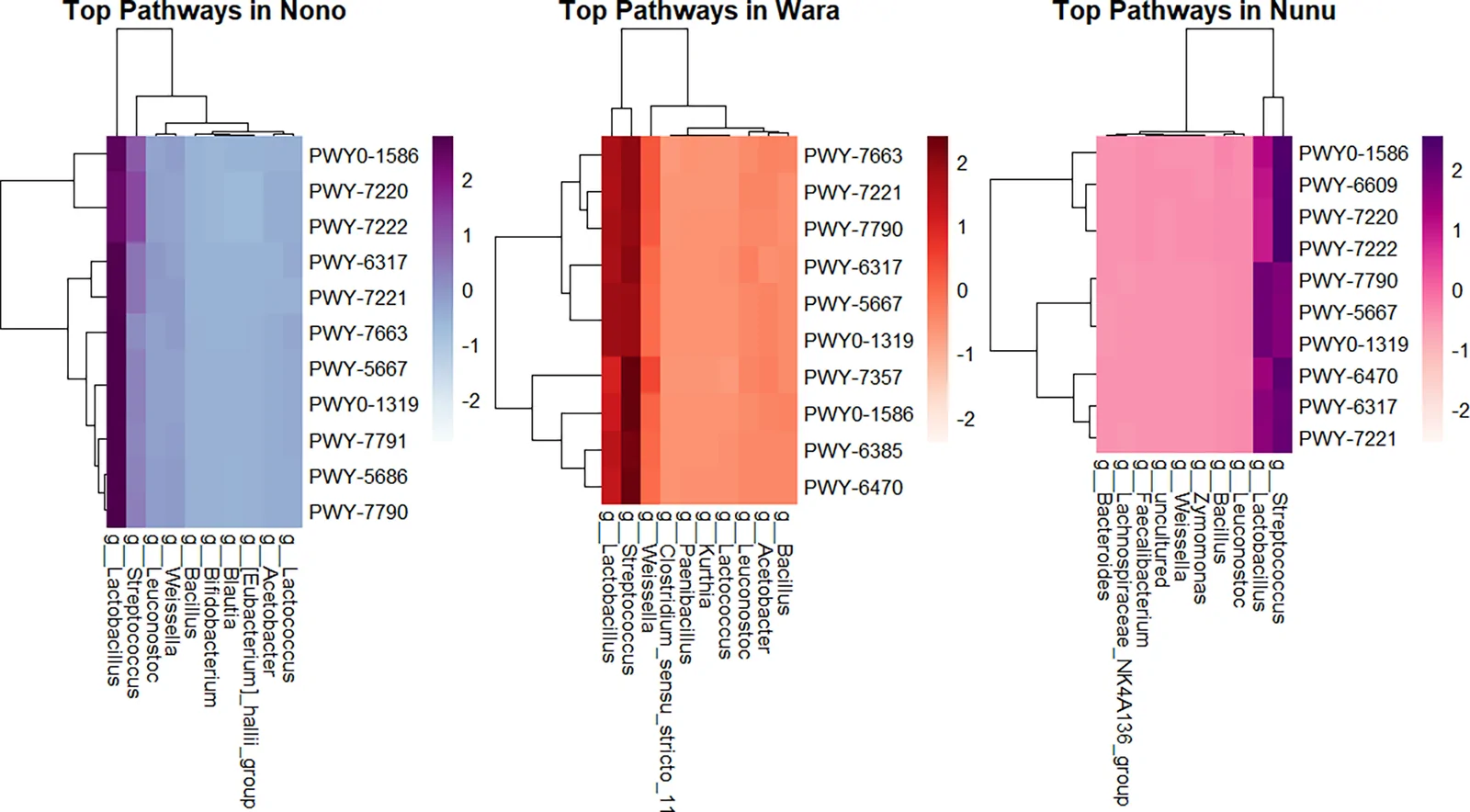
Pathway abundance of the dairy products. Rel_abundance=(abundance of a specific pathway contributed by a genus)/(sum of all pathway abundances across all genera and pathways)×100. PWY-5686=UMP biosynthesis I, PWY-7720=adenosine deoxyribonucleotides *de novo *biosynthesis II, PWY-7222=guanosine deoxyribonucleotides *de novo* biosynthesis II, PWY-7791=UMP biosynthesis III, PWY0−1319=CDP-diacylglycerol biosynthesis II, PWY-5667=CDP-diacylglycerol biosynthesis I, PWY-1586=peptidoglycan maturation (meso-diaminopimelate containing), PWY-7221=guanosine ribonucleotides *de novo* biosynthesis, PWY-6317=d-galactose degradation I, PWY-7663=gondoate biosynthesis (anaerobic), PWY-7791=UMP biosynthesis II. PWY-6470=peptidoglycan biosynthesis V (β-lactam resistance), PWY–7357=thiamine diphosphate formation from pyrithiamine and oxythiamine, PWY-7790=UMP biosynthesis II, PWY-6609=adenine and adenosine salvage III and PWY-6317=d-galactose degradation I*.*

In Wara, *Streptococcus* contributed the most to all the top-represented pathways, gondoate biosynthesis (anaerobic) (PWY-7663), guanosine ribonucleotides *de novo* biosynthesis (PWY-7221), d-galactose degradation I (PWY-6317), peptidoglycan maturation (meso-diaminopimelate containing) (PWY0-1586), peptidoglycan biosynthesis III (PWY-6385) and peptidoglycan biosynthesis V (β-lactam resistance) (PWY-6470) at 36.3, 39.9, 42.7, 46.3, 45.1 and 47.4%, respectively. *Lactobacillus* also contributed substantially, ranging from 24.5 to 38.7% across the top-represented enzyme pathways.

*Streptococcus* contributed the most to some of the top-represented pathways, peptidoglycan maturation (meso-diaminopimelate containing) (PWY0-1586), adenine and adenosine salvage III (PWY-6609), adenosine deoxyribonucleotides *de novo* biosynthesis II (PWY-7220), guanosine deoxyribonucleotides *de novo* biosynthesis II (PWY-7222), peptidoglycan biosynthesis V (β-lactam resistance) (PWY-6470) at 54.8, 61.2, 62.8, 62.8 and 53.3%, respectively. *Lactobacillus* also contributed the most to these pathways, UMP biosynthesis II (PWY-7790), CDP-diacylglycerol biosynthesis I (PWY-5667) and CDP-diacylglycerol biosynthesis II (PWY0-1319) at 46.8, 48.0 and 48.0%, respectively.

### Genus contribution to the enriched pathways in Kwerionik

The top functionally abundant metabolic pathways in the fermentation microbiome of the Kwerionik dairy product are presented in Fig. 11. *Lactobacillus* contributed the most to all the top-represented pathways with the top contributions being peptidoglycan biosynthesis III (PWY-6385), UMP biosynthesis II (PWY-7790), UMP biosynthesis III (PWY-7791), UMP biosynthesis I (PWY-5686), CDP-diacylglycerol biosynthesis I (PWY-5667) and CDP-diacylglycerol biosynthesis II (PWY0-1319) at 41.2, 42.2, 43.1, 40.8, 41.4 and 41.4%, respectively. *Acetobacter* and *Leuconostoc* also contributed substantially, ranging from 15.6to 22.7% and from 12.1 to 17.3%, respectively, to the top-represented pathways.

**Fig. 11.**
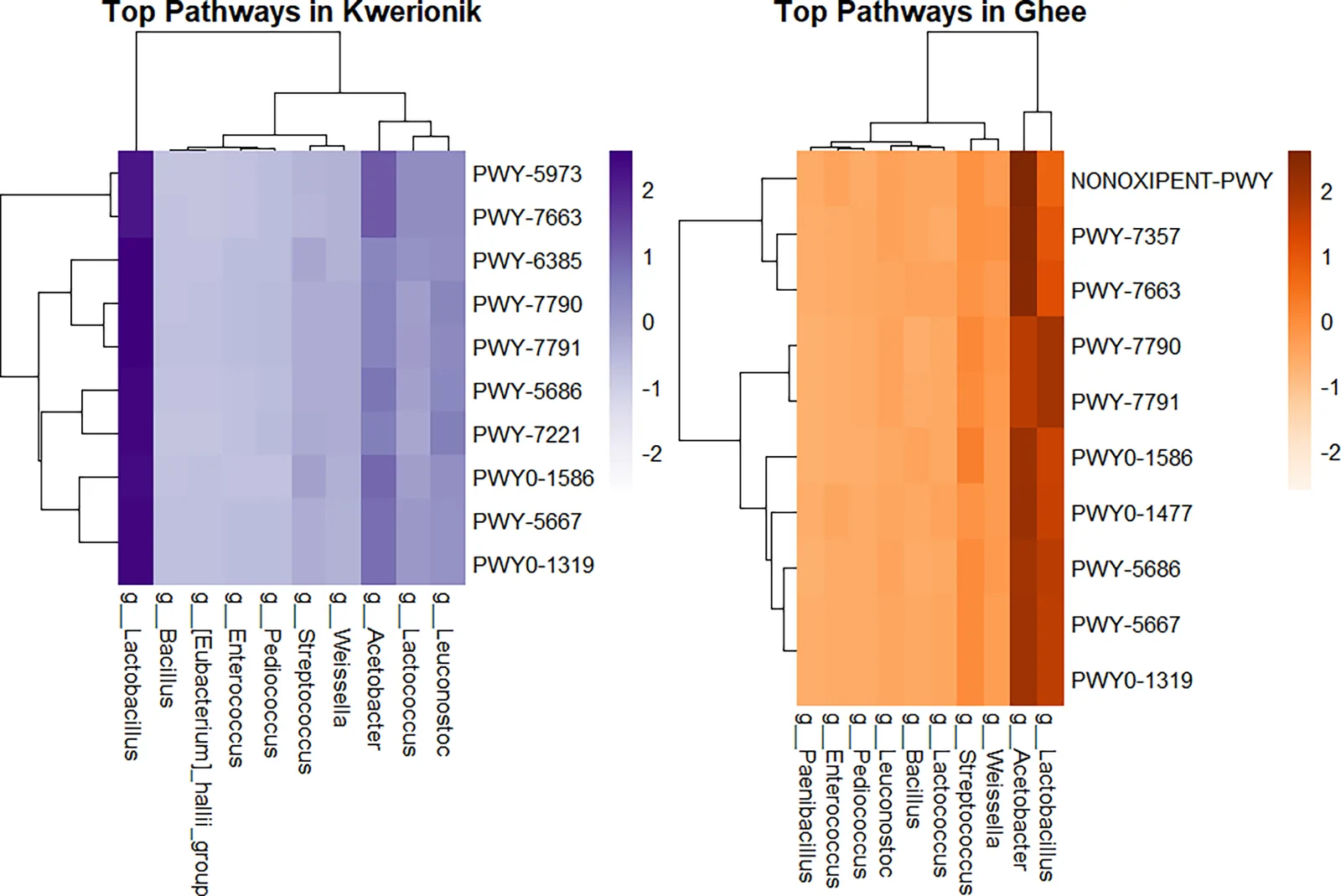
Predicted pathway abundance of the Kwerionik dairy product. Rel_abundance=(abundance of a specific pathway contributed by a genus)/(sum of all pathway abundances across all genera and pathways)×100. PWY-5686=UMP biosynthesis I, PWY-7791=UMP biosynthesis III, PWY0−1319=CDP-diacylglycerol biosynthesis II, PWY-5667=CDP-diacylglycerol biosynthesis I, PWY-1586=peptidoglycan maturation (meso-diaminopimelate containing), PWY-7790=UMP biosynthesis II, PWY-7663=gondoate biosynthesis (anaerobic), PWY-7221=guanosine ribonucleotides *de novo* biosynthesis, PWY-5973=*cis*-vaccenate biosynthesis, NONOXIPENT-PWY=pentose phosphate pathway (non-oxidative branch) I and PWY-7357=thiamine diphosphate formation from pyrithiamine and oxythiamine.

In Ghee, *Acetobacter* contributed the most to some of the top-represented pathways, pentose phosphate pathway (non-oxidative branch) I (NONOXIPENT-PWY), gondoate biosynthesis (anaerobic) (PWY-7663), peptidoglycan maturation (meso-diaminopimelate containing) (PWY0-1586), ethanolamine utilization (PWY0-1477) and CDP-diacylglycerol biosynthesis II (PWY0-1319) at 50.9, 43.1, 37.6, 41.2 and 39.1%, respectively. *Lactobacillus* contributed the most to UMP biosynthesis II (PWY-7790) and UMP biosynthesis III (PWY-7791) pathways at 32.0 and 32.8%, respectively.

## Discussion

This study investigated the fermentation microbiome of selected dairy products from Nigeria, Ghana and Uganda. Nono, Nunu and Kwerionik are fermented milk drinks, whilst Wara is a soft cheese and Ghee is milk butter. Previous studies on microbial communities have primarily relied on conventional culture-dependent microbiological methods [26], which are limited in scope due to the inability to culture many bacterial species. To overcome this limitation, we used a 16S rRNA-targeted amplicon sequencing approach, enabling comprehensive characterization of the fermentation microbiome.

The 485 ASVs were classified as 12 phyla, 16 orders, 50 classes, 85 families and 177 genera across the five samples on average (Nono, Wara, Kwerionik, Ghee and Nunu). At the genus taxon level, 21 ASVs were classified as uncultured/unidentified, which accounted for 1,521 sequence reads across all samples. *Lactobacillus* predominates mainly in Nono at 57%, whereas Nunu and Wara shared *Streptococcus* and *Lactobacillus* at ∼30% each on average (Fig. 1). This indicates the dominance of LAB in these three dairy products. However, in Ghee and Kwerionik, LAB (*Lactobacillus*) shares dominance with *Acetobacter,* a non-*Lactobacillus* (LAB) species. These observations are consistent with studies on microbiota classification of dairy products in Africa [5, 27–29].

Enzyme activity, KO and pathways were used to categorize the functional analysis. Relative abundance was calculated to draw focus on the top 10 microbiota. First, all dairy products were compared to assess which had greater functional abundance (Fig. 3). Varied functional capacities were observed among the dairy products, with Ghee and Nunu showing more pronounced functionality. In the analysed sample, Nunu exhibited the highest predicted abundance for enzymes involved in critical cellular processes. These include site-specific DNA-methyltransferase (adenine-specific) (EC 2.1.1.72), DNA helicases (EC 3.6.4.12), DNA-directed RNA polymerase (EC 2.7.7.6), DNA-directed DNA polymerase (EC 2.7.7.7), histidine kinase (EC 2.7.13.3) and serine-type d-Ala-d-Ala carboxypeptidase (EC 3.4.16.4). These enzymes have been shown to play essential roles in DNA replication, repair, transcriptional regulation, signal transduction and peptidoglycan remodelling. This suggests that the microbial community in Nunu is highly active in genetic information processing and cell envelope biosynthesis, such as rapid growth, stress response or horizontal gene transfer in the fermentation environment [30–32]. Specifically, the presence of DNA helicases and polymerases is indicative of robust microbial growth and replication potential [30], while histidine kinases point to enhanced signal transduction and environmental sensing abilities among Nunu’s microbial taxa [33]. d-Ala-d-Ala carboxypeptidase, associated with cell wall synthesis, further supports the idea of a metabolically active and structurally adaptive microbiome in Nunu.

In contrast, Ghee showed the highest predicted abundance for 6-phospho-β-glucosidase (EC 3.2.1.86), ABC-type polar amino acid transporter (EC 3.6.3.21), asparaginyl-tRNA synthase (glutamine-hydrolyzing) (EC 6.3.5.6) and glutaminyl-tRNA synthase (EC 6.3.5.7). These enzymes are essential for carbohydrate catabolism, amino acid transport and protein biosynthesis. The high abundance of 6-phospho-β-glucosidase, which hydrolyzes phosphorylated β-glucosides, suggests an enhanced ability to break down complex sugars, possibly linked to residual milk sugars in clarified butter, such as Ghee [34]. Similarly, elevated ABC transporters and aminoacyl-tRNA synthetases highlight efficient amino acid uptake and protein translation capacity, reflecting microbial adaptation to Ghee’s nutrient profile and storage environment [35].

After comparing the functional potential of the five dairy products, this study further examined the individual products to identify the taxa driving the predicted functions. Consistently across the individual products, LAB, specifically *Streptococcus* and *Lactobacillus* and non-LAB, *Acetobacter*, contributed the most to the functional potential of the fermentation microbiome. The results point to convergence and divergence among the dominant taxa in their contributions to specific enzymatic activities. Thus, offering insights into the microbial ecology and metabolic specialization that is inherent in each sample.

Across the analysed samples, *Lactobacillus* and *Streptococcus* emerged as central contributors to core enzymatic functions, yet their dominance and inferred roles varied considerably across products. In Nunu, *Streptococcus* was the most dominant taxon contributing to the most abundant enzyme activities, including *N*-acetylmuramoyl-l-alanine amidase, histidine kinase and ABC-type polar-amino-acid transporters (Fig. 6). These enzymes are associated with cell wall metabolism, nutrient uptake and environmental sensing. These functions are critical for bacterial proliferation and survival in milk-based fermentations [33, 35, 36]. Furthermore, within the same product, *Lactobacillus* contributed more to inferred DNA helicase and pseudouridine synthase activities. This collaborative division of functional contributions suggests a metabolic symbiosis between *Streptococcus* and *Lactobacillus*, with the former regulating rapid environmental adaptation and the latter supporting transcriptional and translational stability [37, 38]. A similar symbiotic dynamic was observed in Wara, where *Streptococcus* again dominated, accounting for over 70% of *N*-acetylmuramoyl-l-alanine amidase activity and over 58% of histidine kinase and amino acid transporter activity. These findings suggest a conserved functional potential for *Streptococcus* across products in regulating structural and adaptive pathways. However, in Wara, *Lactobacillus* had a more limited functional potential, contributing to DNA helicase activity (39.4%). This suggests a functional potential restricted to DNA replication and repair. This contrast highlights product-specific functional stratification. This could be influenced by differences in substrate composition and the type of fermentation employed by product producers [39, 40].

Compared to Nunu and Wara, Kwerionik showed a more balanced functional contribution from *Lactobacillus, Acetobacter* and *Lactococcus* (Fig. 7). *Lactobacillus* was the primary contributor to most of the inferred enzyme activities, including aminoacyl-tRNA synthases and DNA helicase. LAB, such as *Lactobacillus*, are multipurpose fermenters that can break down carbohydrates and produce proteins [41].

Perhaps the most taxonomically distinct profile was predicted in Ghee, where *Acetobacter* dominated the enzymatic landscape. It contributed more than 90% of the inferred NADH: ubiquinone reductase activity. *Acetobacter* was also the predominant contributor to nucleic acid-processing and signal transduction enzymes, such as RNA helicases and DNA-directed polymerases. The predominance of *Acetobacter* in Ghee likely reflects the product’s unique properties, such as an oxidative and high pH environment, which favours acid-tolerant aerobes [42, 43]. The dominance of *Streptococcus* in Nunu and Wara may point to early-stage, rapid fermentations, whereas the functional potential in *Acetobacter* within Ghee indicates later-stage oxidative transformations. Such microbial interactions influencing the fermentation microbiome have implications for flavour development, texture and even probiotic potential, as different taxa contribute unique bioactive metabolites and enzymatic properties [28, 44, 45].

Across the analysed samples, all products demonstrated strong enrichment for KO genes. These are genes associated with ABC transport systems, aa uptake and sugar regulation. Their functional pathways are well recognized as metabolic signatures of milk fermented microbiomes and are crucial for nutrient acquisition, acid production and microbial competition. *Lactobacillus* contributed most to the functional potential of Nono’s fermentation microbiome. *Streptococcus* dominated Nunu’s functional potential by contributing more than 60% to most key KO genes. Specifically, ABC transporter KO genes (Fig. 8). However, in the Wara dairy product, *Streptococcus* contributed in a more balanced manner. *Lactobacillus* is documented for its role in genomic capacity for peptide and amino acid transport, as well as its robust proteolytic system in dairy environments [46–48]. Ghee revealed a unique taxonomic-functional signature (Fig. 9). While *Lactobacillus* was dominant across most ABC transporter genes, *Acetobacter* was the key contributor to K02014 (putative ABC transporter permease). This points to Ghee’s lipid-rich and aerated environment, which favours *Acetobacter* and its potential role in horizontal gene transfer [49, 50].

The predicted metabolic pathways across the analysed samples showed that taxa contributed similarly to certain products and differently to others. However, collectively, metabolic pathways showed functional potential, such as nucleotide biosynthesis and energy metabolism dominating the fermentation microbiome. In all these instances, the taxa *Streptococcus, Lactobacillus* and *Acetobacter* contributed most across the five products.

In Nono, *Lactobacillus* emerged as the dominant contributor across all major predicted pathways, particularly those involved in nucleotide biosynthesis (e.g. UMP biosynthesis I–III, PWY-5686, PWY-7790, PWY-7791) and membrane phospholipid formation (CDP-diacylglycerol biosynthesis I and II) (Fig. 10). *Lactobacillus* contribution in this context exceeded 60%, thus indicating an efficient fermentation environment driven by a single genus. *Streptococcus*, while less dominant, also contributed complementary support, particularly in peptidoglycan maturation and amino acid biosynthesis, indicative of a synergistic role as LAB [3, 6, 51].

A contrast emerged within Wara, where *Streptococcus* tended to dominate in contributing most to the predicted functional pathways. Especially those involved in cell wall biosynthesis and resistance pathways, such as peptidoglycan maturation (PWY0-1586) and β-lactam resistance-associated biosynthesis (PWY-6470). This may indicate a stress response to microbial competition, in which structural reinforcement becomes essential. *Lactobacillus* also showed meaningful contributions (25–39%) across many of the same pathways. This highlights its supporting metabolic role, consistent with LAB working together in a fermentation environment [2, 6, 52].

Another significant contrast was observed with Ghee, where *Acetobacter* dominates, contributing chiefly to oxidative and lipid metabolism (Fig. 11). The metabolic network of Ghee suggests a significant shift from the LAB-dominated profiles observed in the other products. These pathways include the NONOXIPENT-PWY, ethanolamine utilization and unsaturated fatty acid biosynthesis. They usually play functions pointing to a microbiome adapted to oxidative, low-moisture environments, which are traits characteristic of Ghee’s post-processing conditions [53]. *Lactobacillus* played a secondary role in UMP biosynthesis; the dominance of *Acetobacter* aligns with previous findings in lipid-rich or post-pasteurized fermented products [49, 50, 54].

In Nunu, *Streptococcus* and *Lactobacillus* co-dominated in terms of their contribution. *Streptococcus* dominated nucleotide *de novo* and salvage pathways (e.g. PWY-7220, PWY-6609), peptidoglycan biosynthesis and β-lactam resistance, while *Lactobacillus* contributed heavily to UMP biosynthesis and membrane lipid synthesis. This suggests a microbial ecosystem with functional specialization, in which different genera assume distinct metabolic roles to optimize overall community performance [6, 55]. The taxonomic contributors to the core biosynthetic and structural pathways of traditionally fermented dairy products differ, reflecting the ecological adaptations and processing conditions specific to each product. The safety, stability and sensory quality of fermented foods are shaped by microbial functional dynamics, not just composition, as this study demonstrates.

In conclusion, these findings suggest that traditional dairy fermentations are microbial ecosystems that are dynamic, resilient and deeply intertwined. Inferring the functional ecology of these microbial communities not only preserves food heritage for scientific documentation. It also lays the groundwork for microbiome-informed innovations in food biotechnology, probiotic development and fermentation standardization. There is, therefore, a need for confirmatory studies, which should involve a shotgun sequencing approach.

## Study limitation

A key limitation of this study is that each dairy product was represented by a single sequencing dataset obtained from publicly available sources. As a result, the analysis lacks biological replication, limiting the ability to draw statistical inferences or generalize findings across product types. Observed differences in taxonomic composition and predicted functional profiles may, therefore, reflect sample-specific variation, including stochastic fermentation dynamics, sample handling or sequencing platform variability. Consequently, the results presented here should be interpreted as descriptive and hypothesis-generating rather than definitive representations of each product category. Future studies incorporating shotgun sequencing with replicated sampling across multiple production batches and locations will be essential to validate and extend these findings.

## Abbreviations

ABC: ATP-binding cassette
ASV: amplicon sequence variant
DADA2: Divisive Amplicon Denoising Algorithm 2
KEGG: Kyoto Encyclopedia of Genes and Genomes
KO: KEGG Orthology
LAB: lactic acid bacteria
NONOXIPENT-PWY: non-oxidative pentose phosphate pathway
NSTI: Nearest Sequenced Taxon Index
PICRUSt2: Phylogenetic Investigation of Communities by Reconstruction of Unobserved States 2
QIIME2: Quantitative Insights into Microbial Ecology 2
